# Using diffusion MRI for measuring the temperature of cerebrospinal fluid within the lateral ventricles

**DOI:** 10.1111/j.1651-2227.2009.01528.x

**Published:** 2010-02

**Authors:** LR Kozak, M Bango, M Szabo, G Rudas, Z Vidnyanszky, Z Nagy

**Affiliations:** 1 MR Research Center, Szentagothai J. Knowledge Center, Semmelweis UniversityBudapest, Hungary; 2 First Department of Pediatrics, Semmelweis UniversityBudapest, Hungary; 3 Neurobionics Research Group, Hungarian Academy of Sciences – Pazmany Peter Catholic University, Semmelweis UniversityBudapest, Hungary; 4 Wellcome Trust Centre for Neuroimaging, UCL Institute of NeurologyLondon, UK; 5 Department of Woman and Child Health, Neonatal Unit, Karolinska University HospitalStockholm, Sweden

**Keywords:** Asphyxia, Diffusion, Magnetic resonance imaging, Temperature, Thermometry

## Abstract

**Aim:**

Hypothermia is often induced to reduce brain injury in newborns, following perinatal hypoxic–ischaemic events, and in adults following traumatic brain injury, stroke or cardiac arrest. We aimed to devise a method, based on diffusion-weighted MRI, to measure non-invasively the temperature of the cerebrospinal fluid in the lateral ventricles.

**Methods:**

The well-known temperature dependence of the water diffusion constant was used for the estimation of temperature. We carried out diffusion MRI measurements on a 3T Philips Achieva Scanner involving phantoms (filled with water or artificial cerebrospinal fluid while slowly cooling from 41 to 32°C) and healthy adult volunteers.

**Results:**

The estimated temperature of water phantoms followed that measured using a mercury thermometer, but the estimates for artificial cerebrospinal fluid were 1.04°C lower. After correcting for this systematic difference, the estimated temperature within the lateral ventricles of volunteers was 39.9°C. Using diffusion directions less sensitive to cerebrospinal fluid flow, it was 37.7°C, which was in agreement with the literature.

**Conclusion:**

Although further improvements are needed, measuring the temperature within the lateral ventricles using diffusion MRI is a viable method that may be useful for clinical applications. We introduced the method, identified sources of error and offered remedies for each.

## Introduction

Severe deviations from the normal physiological temperature present serious risk to the body. Hyperthermia can lead to convulsion in infants and worsen the neuronal damage that otherwise would ensue from primary or secondary injury to the brain (1). A reduction in the body temperature can also be life-threatening, however, because of the accompanied reduction in biochemical reaction rates, mild hypothermia has been found to be of protective value.

Recently, much attention has been paid to its neuroprotective effects (2). In 1989, Busto et al. reported a reduction in damage to the C1 region of the rat hippocampus after a period of mild cooling (3). Later, Thoresen et al. found neuroprotective effects in a neonatal animal model of hypoxia (4). Since then induced hypothermia has been used in newborn infants with hypoxic–ischaemic encephalopathy (5), and in adults after stroke (6), or traumatic brain injury (7). However, the temperature is usually monitored rectally, orally or within the ear during the course of the hypothermic period, giving only indirect indication of the actual temperature of the brain itself.

MR imaging allows the estimation of the diffusion constant of a sample (8,9). Based on the temperature dependence of the water diffusion constant (10), we propose to estimate the temperature of the cerebrospinal fluid (CSF) within the lateral ventricles. Similar methods have been implemented in a gel phantom (11), in the muscle tissue (12,13) and in cadavers (14) previously. While other MRI and MR spectroscopy parameters are also temperature-dependent (15,16), our proposed method may be advantageous because diffusion tensor imaging (DTI) is often performed in the current clinical and research protocols. Although, this does not require extra scanning time and allows for retrospective temperature estimates, we identified several shortcomings. We offer remedies to reduce their effects and also discuss how the protocol could be optimized if further imaging time was available.

## Methods

### Data acquisition

All images were collected on a 3T Philips Achieva Scanner (Philips Medical Systems, Best, The Netherlands) with an 8-channel head coil. A single-shot EPI sequence was employed, including 32 images with non-collinear diffusion directions and a b-value of 800 s/mm^2^ as well as a single b = 0 s/mm^2^ reference image. The following imaging parameters were used for both the phantom and *in vivo* measurements: FOV = 190 mm, imaging matrix = 128 × 127, TR = 8000 ms, TE 90 ms, 20 slices with thickness = 1.5 mm (1.5 mm gap).

### Phantom experiments

The phantoms were 12 × 6 × 6 cm rectangular high-density polyethylene bottles. One bottle (Bottle 1) was filled with artificial cerebrospinal fluid (ACSF, for chemical composition see Table 1) (17) and two other identical bottles (Bottle 2 and 3) were filled with the distilled water that was used for the ACSF preparation. All bottles were heated to 41.5°C in a water bath. Bottles 1 and 2 were placed side-by-side. Bottle 3 was placed on top of them and used only for monitoring the temperature manually with a mercury thermometer with 0.1°C grating, built by Lombik Kft (Budapest, Hungary) and having a measurement uncertainty of 0.1°C, as calibrated by the ISO/IEC 17025 certified (NAT-2-0133/2004) KVALIFIK Kft (Budapest, Hungary) using the Hom-Kalelj-17 method.

**Table 1:** Composition of the artificial cerebrospinal fluid

Chemical	Concentration
Na+	151 (mm)
K+	3.0 (mm)
Mg^2+^	1.0 (mm)
Ca^2+^	1.4 (mm)
Cl^−^	133 (mm)
HCO^3−^	25.8 (mm)
Glucose	4.2 (mm)
Amino acids	0.8 (mm)
Protein	250 (mg/L)

To prevent rapid cooling, the bottles were bundled in blankets. The manual temperature measurements were carried out just after the acquisition of diffusion data. For the manual measurement, the scanner couch was slightly retracted, without moving the bottles within the head-coil. Subsequently, the scanner couch was driven back to the previous position and the whole arrangement was left to settle for at least 15 min before the next set of images were acquired with the isocentre being in the middle of the centre slice. Using this protocol, the sequential manual temperature measurements were taken 25 min apart.

Data were also collected 12 times consecutively at room temperature (having distilled water in each phantom) to assess the stability and reproducibility of the method, and also to compare values between ROIs placed on the left and the right.

### Gradient calibration experiment

Further data were collected in a separate session for b-value correction measuring ADC values in +*X*, −*X*, +*Y*, −*Y*, +*Z* and −*Z* direction (18). For this purpose, a sufficiently large phantom was used to cover both ROIs of the previous experiments.

### Human experiments

To assess the reproducibility and feasibility of the method *in vivo*, five volunteers (mean age 32.6 ± 5.2 years) were involved. Four of them (one woman) underwent a single scan each, while a single, male volunteer was imaged three times. The local ethics board approved the involvement of human volunteers in this study, each of whom signed an informed consent.

### Data processing

All processing of the collected MR images was performed in the Matlab 7.1 (MathWorks Inc., Natick, MA, USA).

The diffusion constant was calculated voxel-wise for each of the 32 diffusion-weighted images. Subsequently, a temperature map was created corresponding to each of the 32 diffusion-weighted images according to (19) 
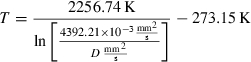
 where T is in units of Celsius. Finally, a mean value was calculated for each voxel.

In the case of phantoms either a 6 × 6 voxels region of interest (ROI) was selected in the centre slice, or a 12 × 12 voxels ROI spanning the 12 slices closest to the isocentre (the latter to better simulate *in vivo* circumstances because the lateral ventricles span several slices).

For the human volunteers, the results were only interpreted within the lateral ventricles. To delineate the lateral ventricles, the b = 0 s/mm^2^ reference image was thresholded to create a binary mask.

To determine the possible effect of CSF pulsation and/or flow, we calculated the mean direction of the diffusion direction vectors that contributed to the asymmetric tail of the temperature distributions. The mean direction was colour-coded in RGB colour space, with red, green and blue representing, respectively, the *x*, *y* and *z* components of the diffusion encoding direction. Based on this analysis, we also calculated temperature estimates using the nine diffusion directions falling within a cone of 45° apical angle with its axis coinciding the patients’ left–right axis. These directions were almost perpendicular to the axes of the lateral ventricles and least likely to produce erroneous results.

In a separate analysis, we also investigated the usefulness of setting absolute temperature thresholds for eliminating outliers in the skewed distribution to correct the *in vivo* temperature estimates. For this purpose, we parametrically varied lower and upper temperature thresholds.

### Statistical methods

Linear regression analysis was performed to evaluate correlation between the measured and the estimated temperatures. Paired Student’s *t*-test was employed to compare the temperature estimates between ROIs placed on the left or the right. Kolmogorov–Smirnov test was used to compare the distributions of temperature values obtained from the volunteer who underwent three scans. In each case, a p-value of 0.05 was considered as the level of statistical significance.

## Results

### Phantom experiments

**Figure 1:**
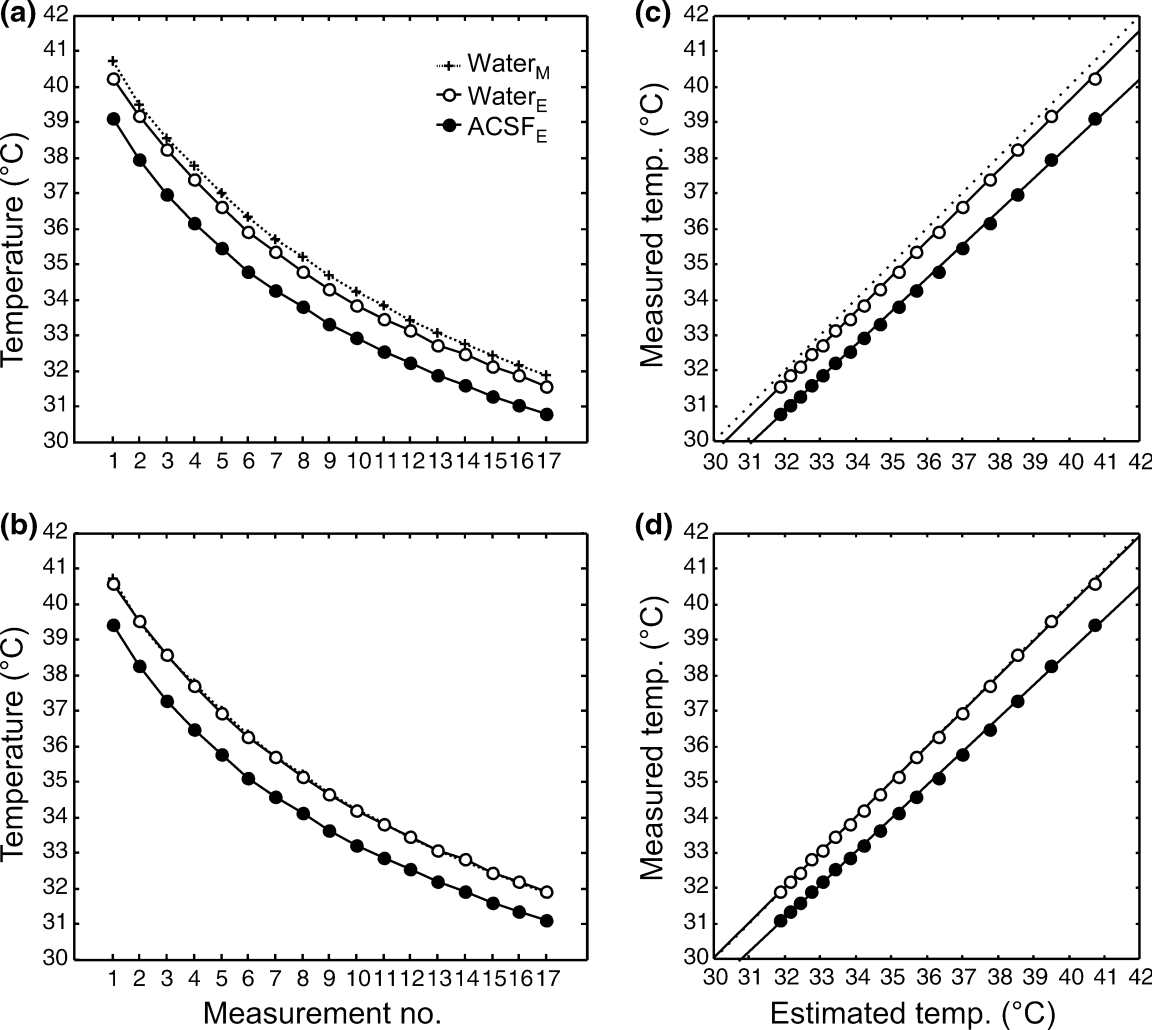
Results of the phantom measurements. The temperature estimates of the slowly cooled phantoms using MRI (a) without gradient calibration and (b) after b-value correction. The estimated temperatures plotted against the measured temperatures (c) without gradient calibration and (d) after b-value correction. Each data point represents the mean temperature in a VOI, which consisted of 12 voxel by 12 voxel ROIs drawn 12 consecutive slices. The subscripts M and E stand for temperature measured with mercury thermometer or estimated with diffusion imaging respectively. The measurements as shown on the *x*-axes of panels a and b were 25 min apart. The solid lines represent linear regression and the dotted line is the line of identity in panels c and d.

Figure 1 displays the results of the measurements on slowly cooling phantoms that were filled either with pure water or ACSF. The estimated measurement within the water phantom (Bottle 2) corresponded well with that measured in Bottle 3 using the mercury thermometer, although it was consistently lower. This systematic difference was −0.43 ± 0.1°C for the ROI at the isocentre and −0.37 ± 0.1°C for the volume of interest (VOI) spanning 12 slices. The estimated temperature within the phantom filled with ACSF was also consistently lower. In this case, the systematic difference was −1.45 ± 0.3°C for the smaller ROI and −1.37 ± 0.2°C for the VOI.

The difference between the temperature estimated from ACSF and measured in water was expected because the macromolecules in solution can hinder water diffusion. The slight offset of the estimated temperature within the water phantom, however, was unforeseen. Upon further investigation, we found that a miscalibration of b-values accounted fully for this discrepancy. After applying b-value correction (18), the previously observed −0.43°C systematic difference between the estimated and measured water temperatures was reduced to below experimental errors (−0.02 ± 0.05°C for the VOI). Accordingly, the observed systematic bias for ACSF also decreased from −1.37°C to −1.04.

We investigated the relationship between the measured and estimated temperatures and found strong linear correlations. Before and after b-value correction, the regression parameters were *Y* = 0.09 + 0.98*X vs. *Y* = 0.35 + 0.99*X for water and *Y* = 0.98 + 0.93*X vs. *Y* = 1.23 + 0.94*X for ACSF respectively (r^2^ = 1, p < 0.0001, for all regressions).

The 12 consecutive measurements at a constant temperature indicated an excellent reproducibility for the method. The standard deviation of the temperature estimates was 0.12°C in both ROIs, and we found no statistically significant difference between the measurements taken from ROIs placed on either side (NS, p > 0.26, paired Student’s *t*-test).

### Human experiments

**Figure 2:**
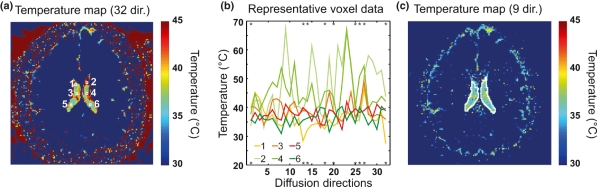
Estimating CSF temperature in the lateral ventricles of a human volunteer. (a) Uncorrected temperature map. Note the artificially high temperatures within the lateral ventricles because of outliers and the artificially low temperature estimates for brain tissue because of the restricted diffusion. (b) Display of all the 32 diffusion constant estimates from six representative voxels (marked with white squares in panel a). The asterisks represent the nine directions found to be least prone artefacts. (c) Temperature map where only the directions marked on panel b were averaged for each voxel within the lateral ventricles. The mean estimated temperature within the lateral ventricles is 37.9°C in this slice.

The temperature estimates of three consecutive measurements on the healthy adult volunteer were 40.5 ± 0.2°C (after correcting for the systematic difference observed during the phantom experiments, see Fig. 2a). There was no statistically significant difference between the distribution of temperature estimates among the three measurements (Kolmogorov–Smirnov test), further supporting the robustness of the method. The mean of the estimated temperature of the four additional volunteers was 39.4 ± 0.9°C (the mean of all estimates being 39.9 ± 0.9°C). These values were higher than expected from previous estimates (16,20).

**Figure 3:**
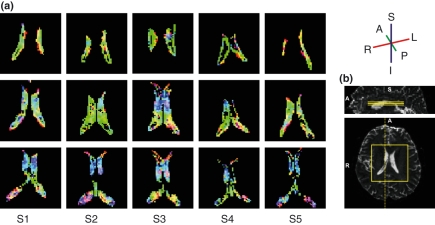
Colour-coded indication of directionality in the excluded data. (a) Mean diffusion direction of data that contributes to the highly asymmetric tail of the distribution of temperature estimates in the five volunteers. Colour coding represents the orientation of the mean diffusion direction according to top of panel b. The mean diffusion-encoding direction follows the curvature of the ventricles. In the more inferior slices (middle and bottom rows), the mean direction has a mainly superior–inferior orientation, especially in the anterior parts of the ventricles, with a marked right–left component in the anterior and posterior horns. In the most superior slice (top row), the mean direction is anterior–posterior. S2 represents the data from the first series of the subject scanned three times. (b) (Top) Indication of the colour scheme where S, I, R, L, A, P stand for superior, inferior, right, left, anterior and posterior respectively. (Middle and bottom) Data from S1 are used to indicate approximate orientation and position of the slices shown in panel a from the five volunteers. S1, etc. stand for Subject 1, etc.

We found that the distribution of voxel-wise temperature estimates was highly skewed in volunteers as opposed to the nearly symmetric distributions observed in the phantoms. Moreover, the mean direction of the diffusion direction vectors that contributed to the highly asymmetric tail of the voxel-wise temperature distributions pointed along the curvature of the lateral ventricles (Fig. 3).

Our attempts of implementing outlier rejection based on absolute temperature thresholds introduced threshold-dependent bias (e.g. by setting the lower threshold to 30°C and varying the upper threshold between 44 and 55°C, the mean temperature estimates in volunteers increased by 0.3°C for every 1°C of change in the threshold).

In a separate analysis, the temperature estimates based on the nine diffusion directions that were almost perpendicular to the axes of the ventricles provided physiologically meaningful results (37.9 ± 0.3°C for the consecutively scanned volunteer and 37.6 ± 0.3°C for the others, thus the mean of all measurements being 37.7 ± 0.3°C, Fig. 2c.).

## Discussion

We have investigated the possibility of using a common DTI dataset that is often collected as part of imaging protocols to measure the temperature in the lateral ventricles of a human brain. As the CSF is in direct contact with the brain tissue, knowledge of its temperature is expected to provide a better estimate of brain temperature than measurements made external to the skull.

Using MR methods for the measurement of temperature is not a new idea. Several MR parameters show temperature dependence, such as proton resonance frequency (15), the chemical shift of water (16), diffusion (11), etc. A theoretical comparison of several methods has been made previously (21). In particular, a study has been presented recently on the application of spectroscopic methods to the population of newborn infants (22).

Diffusion imaging is difficult to use *in vivo* because the diffusion constant of water in tissue is significantly reduced and is directionally variable depending on the tissue composition (21). Previously, Le Bihan et al. (11) have demonstrated the applicability of the diffusion constant in temperature mapping; however, their method depends on a reference measurement at a known temperature and they only applied it *in vitro*. Tofts et al. (14) used diffusion MRI to measure CSF temperature in cadavers, however, their temperature estimates were made far from the physiological range. *In vivo* diffusion MRI-based temperature measurements have been reported in the muscle tissue (12,13).

By applying the method to images of human adults, we found that the temperatures estimated from 32 diffusion directions were higher than expected based on previous observations (16,20). Considering the source of this effect, the most likely factor is that the CSF in the lateral ventricles is not still; hence, the movement of water is not solely molecular diffusion. Diffusion imaging is sensitive to movement in general and as a result, bulk and pulsatile flow may manifest themselves as artificially high diffusion (see Fig. 3). In a separate analysis temperature estimates based on nine diffusion directions that were within a cone of 45° apical angle with its axis coinciding the patients’ left–right axis yielded results that were comparable with previous results (16,20). Using pulse-triggered acquisitions may also, at least partially, remedy the artefacts caused by pulsatile motion; however, as it extends imaging time, it is less often employed for neonatal and/or acute cases.

Another possibility could be to use automated outlier rejection methods, e.g. by excluding measurements below and/or above some given threshold(s). We investigated this possibility and conclude that such thresholding introduces bias to the temperature estimates. There are other outlier rejection techniques based on statistical principles (23,24), but their usefulness cannot be estimated without measurements in a broad range of *in vivo* CSF temperatures, which could only be warranted by animal experiments. Such investigation would also elucidate whether bulk CSF motion is temperature-dependent, and would provide opportunity to direct invasive temperature measurements as controls for diffusion MRI-based temperature estimation.

Another aspect which needs consideration is that the CSF is not pure water but contains chemicals in solution, which hinder the diffusion of water molecules leading to correspondingly lower values for the estimated temperature (Fig. 1). However, this systematic error is stable over the measured temperature range and hence can be easily corrected for. The solution used in these experiments was mimicking the chemical composition of the CSF of a healthy adult, which may differ from that of an infant shortly after a hypoxic–ischaemic event or that of an adult after a stroke, cardiac arrest or traumatic brain injury.

Although the exact composition of CSF remains unknown in most cases, the pattern of changes are being investigated and described in the literature for various disorders (25–27). Further investigation efforts are required to elucidate the relationship between the chemical properties of CSF and temperature estimates obtained by diffusion MRI. Nevertheless, we believe that, should the chemical composition of CSF in a given patient become available, the methods presented here could easily be adjusted. It is important to stress that in cases of comparing the efficiency of cooling methods, or different approaches to measure temperature, the main variable of interest is likely to be the extent of temperature change and not the absolute temperature per se.

A b-value of 800 s/mm^2^ is considered optimal while imaging the brain tissue of neonates suffering from hypoxic/ischaemic encephalopathy (28). Because these patients represent one of the target groups of the temperature mapping method presented here and because the original aim was to obtain these measurements without additional imaging time, this b-value was used in this study. However, if additional imaging time is allocated or it is decided that DTI data are not needed, the optimal b-value for imaging CSF would be approximately 362 s/mm^2^ (29,30). Further improvements in data quality and possible reduction of imaging time could be obtained if repeated measurements were carried out in a single diffusion encoding direction but supplemented by acceleration–compensation gradients. If acceleration–compensation gradients are not available, decreased acquisition time can also be achieved by collecting data in those directions that are least prone to pulsatile and flow artefacts.

Hypothermia is also used as a neuroprotective method in adults after stroke (6), cardiac arrest (31) or traumatic brain injury (7). In addition, acute fever has adverse effects on the brain tissue (2). Monitoring the temperature of the brain non-invasively would be important in these cases as well.

We have demonstrated the feasibility of using MR diffusion imaging experiments for measuring the temperature and the extent of temperature changes of CSF *in vitro* using phantoms, and provided examples of *in vivo* measurements within the lateral ventricles of the human brain. The method is accurate and precise when applied to temperature difference measurements, which could be the most important application for some clinical studies. However, further work is needed to make the presented method a clinical routine. A systematic evaluation of the effects of CSF composition on temperature estimation is crucial. While the described calibration framework that relates CSF composition to temperature estimates with diffusion MRI would be useful for this, animal experiments and invasive probing over a range of temperatures are also needed for the validation of methodology. Although our results suggest that data acquisition perpendicular to the axis of the ventricles, i.e. in left–right direction, can at least partly remedy the bias caused by bulk CSF flow, data obtained *in vivo* at different temperatures are required to determine the optimal diffusion directions and post-processing parameters.

## Abbreviations

ACSF: artificial cerebrospinal fluid
CSF: cerebrospinal fluid
DTI: diffusion tensor imaging
MR: magnetic resonance
MRI: magnetic resonance imaging
ROI: region of interest
VOI: volume of interest
